# Clinical Significance of an IgM and IgG Test for Diagnosis of Highly Suspected COVID-19

**DOI:** 10.3389/fmed.2021.569266

**Published:** 2021-04-12

**Authors:** Xingwang Jia, Pengjun Zhang, Yaping Tian, Junli Wang, Huadong Zeng, Jun Wang, Jiao Liu, Zeyan Chen, Lijun Zhang, Haihong He, Kunlun He, Yajie Liu

**Affiliations:** ^1^Department of Clinical Laboratory Medicine Center, Shenzhen Hospital, Southern Medical University, Shenzhen, China; ^2^Key Laboratory of Carcinogenesis and Translational Research (Ministry of Education/Beijing), Interventional Therapy Department, Peking University Cancer Hospital and Institute, Beijing, China; ^3^Department of Translational Medicine, Chinese PLA General Hospital, Beijing, China; ^4^Department of Respiratory and Critical Care Medicine, Shenzhen Hospital, Southern Medical University, Shenzhen, China; ^5^Key Laboratory of Ministry of Industry and Information Technology of Biomedical Engineering and Translational Medicine, Chinese PLA General Hospital, Beijing, China; ^6^Department of Neurology, Shenzhen Hospital, Southern Medical University, Shenzhen, China

**Keywords:** COVID-19, nucleic acid test, IgM, IgG, CT scan

## Abstract

**Background:** Nucleic acid detection and CT scanning have been reported in COVID-19 diagnosis. Here, we aimed to investigate the clinical significance of IgM and IgG testing for the diagnosis of highly suspected COVID-19.

**Methods:** A total of 63 patients with suspected COVID-19 were observed, 57 of whom were enrolled (24 males and 33 females). The selection was based on the diagnosis and treatment protocol for COVID-19 (trial Sixth Edition) released by the National Health Commission of the People's Republic of China. Patients were divided into positive and negative groups according to the first nucleic acid results from pharyngeal swab tests. Routine blood tests were detected on the second day after each patient was hospitalized. The remaining serum samples were used for detection of novel coronavirus-specific IgM/IgG antibodies.

**Results:** The rate of COVID-19 nucleic acid positivity was 42.10%. The positive detection rates with a combination of IgM and IgG testing for patients with COVID-19 negative and positive nucleic acid test results were 72.73 and 87.50%, respectively.

**Conclusions:** We report a rapid, simple, and accurate detection method for patients with suspected COVID-19 and for on-site screening for close contacts within the population. IgM and IgG antibody detection can identify COVID-19 after a negative nucleic acid test. Diagnostic accuracy of COVID-19 might be improved by nucleic acid testing in patients with a history of epidemic disease or with clinical symptoms, as well as CT scans when necessary, and serum-specific IgM and IgG antibody testing after the window period.

## Introduction

COVID-19 was named by the World Health Organization on January 12, 2020. Coronaviruses are a large family of viruses that cause colds and more serious diseases (1). COVID-19 is caused by a novel coronavirus strain that had not previously been found in humans. Common signs of infection include respiratory symptoms, fever, shortness of breath, and dyspnea. In severe cases, infection can cause pneumonia, acute respiratory syndrome, kidney failure, and even death. There is currently no specific treatment for COVID-19 (2). However, many symptoms can be managed and must be treated according to the clinical situation of each patient. The main routes of transmission of COVID-19 are respiratory droplets and contact transmission. Aerosol and fecal-oral routes of transmission must be further clarified. Epidemiological investigations have shown that cases can be traced to close contact with individuals with confirmed infection (3, 4).

According to the sixth edition of the diagnostic criteria, COVID-19 cases are divided into two categories: suspected cases and confirmed cases. As of midnight on February 28, 2021, a total of 89,912 confirmed cases had been reported in China; 85,066 cases had been cured, and 4,636 deaths had occurred. COVID-19 exhibited a sudden outbreak worldwide (5). Timely and accurate diagnosis is crucial for detection and patient therapy. However, in clinical practice, detection standards have varied with the rapidly growing awareness of COVID-19. Nucleic acid detection, chest CT, epidemiological history, and clinical manifestations are recognized as the diagnostic basis (6, 7). However, nucleic acid detection has the limitations of operator errors, time consumption, and proneness to contamination. The specificity of CT results is also limited. IgM/IgG antibody detection has the advantage of being simple and easy to perform, and has high sensitivity.

In our study, we aimed to provide a quick, simple, and accurate diagnostic method by evaluating the clinical significance of IgM and IgG for the diagnosis of highly suspected COVID-19 cases.

## Materials and Methods

A total of 63 patients with suspected COVID-19 were observed, 57 of whom were finally enrolled, including 24 males and 33 females who were 2–63 years of age (8). Six patients were excluded because of a lack of serum samples. The characteristic features of the patients are described in Table 1. Selection was performed according to the diagnosis and treatment protocol for COVID-19 (trial sixth edition) released by the National Health Commission of the People's Republic of China. The patients who met the standards for suspected COVID-19 were enrolled, and those who did not were excluded. Suspected cases of COVID-19 were defined according to the presence of at least one of the following clear epidemiological history criteria: (1) a history of travel or residency in Wuhan or the surrounding area, or in communities with COVID-19 cases within 14 days before onset; (2) a history of contact with people with COVID-19 (positive nucleic acid test) within 14 days before onset; (3) a history of contact with patients infected with COVID-19 from Wuhan and surrounding areas, or a history of contact with people with fever or respiratory symptoms from communities with COVID-19; and (4) cluster onset. In addition, patients were required to have the following clinical manifestations: (1) fever and/or respiratory symptoms; (2) imaging features of COVID-19; and (3) normal or diminished white blood cells in early stages of disease and a diminished lymphocyte count. If there was no clear epidemiological history, the above three clinical manifestations were necessary for inclusion. This was a retrospective study approved by the Ethics Committee of Shenzhen Hospital, Southern Medical University (NYSZYYEC20200009). The requirement for informed consent was waived because the data were anonymous. Study participants shared the results in strict accordance with the rules of the Ethics Committee of Shenzhen Hospital, Southern Medical University.

**Table 1:** Characteristics and clinical features of patients with suspected COVID-19.

**Characteristic/clinical features**	***n***	**%**
**Sex**		
Male	24	42.11
Female	33	57.89
**Age in years**		
<18	4	7.02
18–45	43	75.44
46–65	10	17.54
**Clinical symptoms**		
Fever	35	61.4
Cough	27	47.37
Fatigue	5	8.77
Shortness of breath	1	1.75
Asymptomatic	4	7.02
Others (headache, sore throat, diarrhea, and so on)	8	14.04
**Imaging findings**		
Characteristic changes	40	70.18
Normal	17	29.82

### Laboratory Examination

The routine blood parameters were detected on the second day of hospitalization. The remaining serum samples were used for detection of IgM and IgG. Primary screening through nucleic acid amplification from pharyngeal swabs was performed with two kits from six companies (DAAN, Sansure Biotech, BGI, ShangHai ZJ Biotech, Geneodx, and Biogerm) in ~20 hospitals in ShenZhen. The time after SARS-CoV-2 exposure to nucleic acid amplification tests (NAAT) and serological tests are described in Table 2.

**Table 2:** Exposure times for NAAT and serological tests.

**Groups**	**Sample number**	**Exposure time, day**			
		**Mean ± SD**	**Range**	**Median (25%, 75%)**	***S-W*-test**
**NAAT**					
All	43	12.86 ± 9.94	1–34	12 (3, 20)	*P* = 0.0041
Positive	18	10.28 ± 7.15	1–34	9 (3, 17.5)	*P* = 0.0560*
Negative	25	14.72 ± 12.31	1–21	14 (3.5, 24.5)	*P* = 0.0301
**Serological test**					
All	43	23.21 ± 8.48	6–39	24 (17, 29)	*P* = 0.4585*
Positive	34	22.82 ± 7.93	10–39	24 (17.75, 29)	*P* = 0.4670*
Negative	9	24.67 ± 10.70	6–39	26 (14.5, 35.5)	*P* = 0.5789*

* *P > 0.05 means the data are normally distributed. Nucleic acid amplification tests, NAAT*.

Most serum samples were obtained 2 weeks after virus exposure; there was only 1 in 6 days and 4 within 2 weeks. The rest of the serum samples were obtained between 14 and 39 days, which is a good detection window period for IgM/IgG (9, 10). A COVID IgM/IgG antibody kit, which was sent to BIMT (Beijing Institute of Medical Device Testing) for product verification, was used with a Time-Resolved Immunofluorescence Analyzer to perform fluorescence immunochromatographic assays (Beijing Diagreat Biotechnologies Co., Ltd., Lot: 20200214, Beijing, China). The procedures of nucleic acid, IgM, and IgG detection were performed strictly according to the manufacturer's manual. A total of 242 healthy people without related diseases were tested, and the values were measured. The 95% confidence intervals for IgM and IgG were 0.44–0.88 U/L and 0.50–1.02 U/L, respectively. These results provided by Beijing Diagreat Biotechnologies Co., Ltd. suggested that the cutoffs for IgM and IgG were 0.88 and 1.02 U/L.

### Data Analysis

Statistical analyses were performed in statistical analysis system software SPSS 19.0. Count data are expressed as percentages. The Shapiro–Wilk normality test was used to evaluate whether the data were normally distributed. Normally and non-normally distributed data are presented as mean ± SD and medians (25th percentile and 75th percentile). Non-parametric tests and two-sided χ^2^-tests were used to compare the differences between groups, and a *P*-value < 0.05 was considered statistically significant.

## Results

### Clinical Characteristics and COVID-19 Nucleic Acid Testing

According to the diagnostic standards for suspected COVID-19, 57 patients were enrolled in our study. All 57 patients underwent three nucleic acid tests, and each time, the results were confirmed with two COVID-19 nucleic acid test kits. Among the 57 patients, 24 patients had a positive nucleic acid test, and 33 patients had a negative nucleic acid test the first time, and all 57 patients had a negative nucleic acid test the second and third times. The positivity rate of COVID-19 nucleic acid testing in the 57 suspected COVID-19 cases was 42.10%.

### IgM and IgG Single Detection of COVID-19

**Figure 1:**
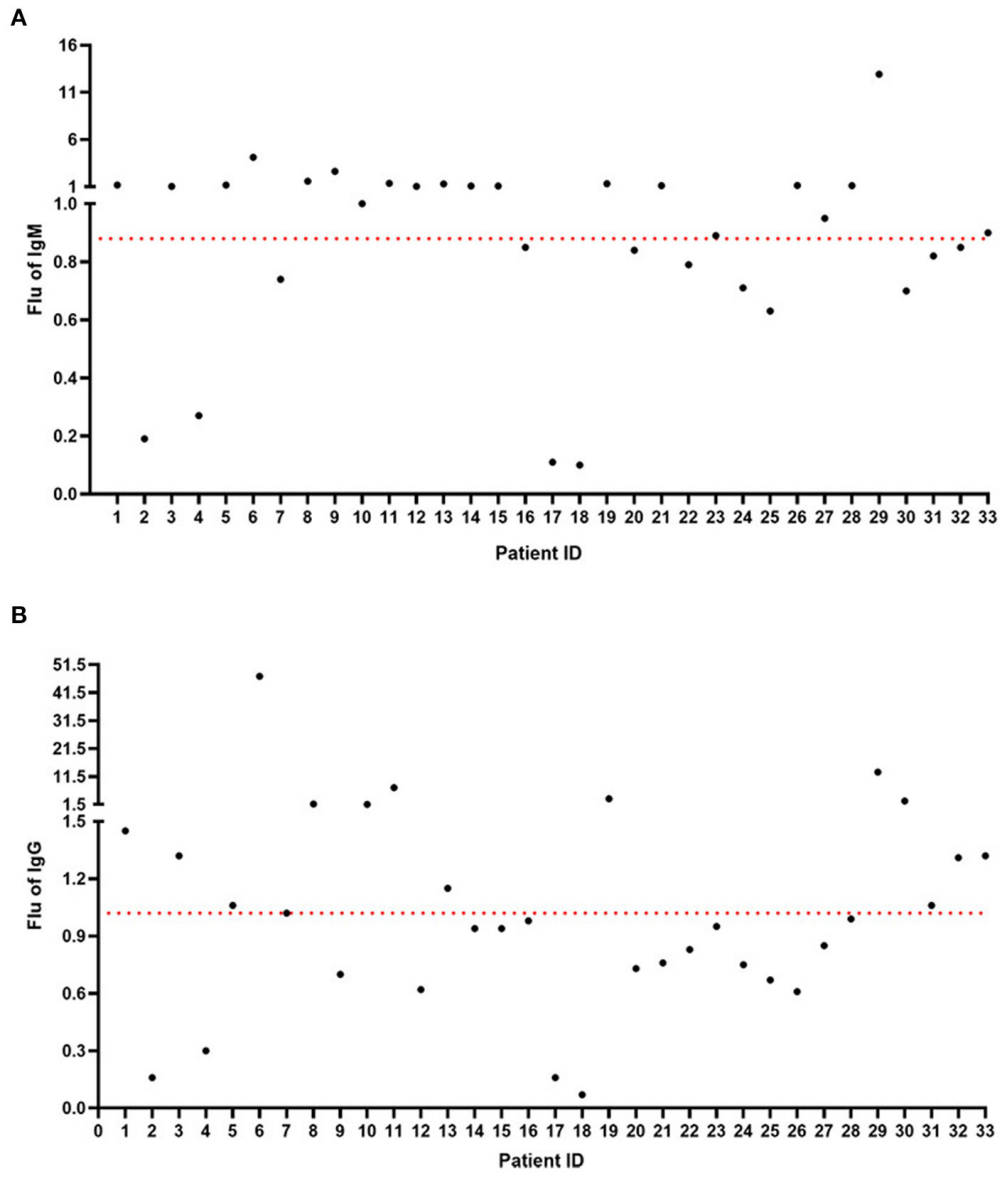
IgM and IgG detection among the 33 patients with negative COVID-19 nucleic acid results. **(A)** The IgM-value of 20 patients was more than 0.88 U/L. **(B)** The IgG-value of 15 patients was more than 1.02 U/L.

**Figure 2:**
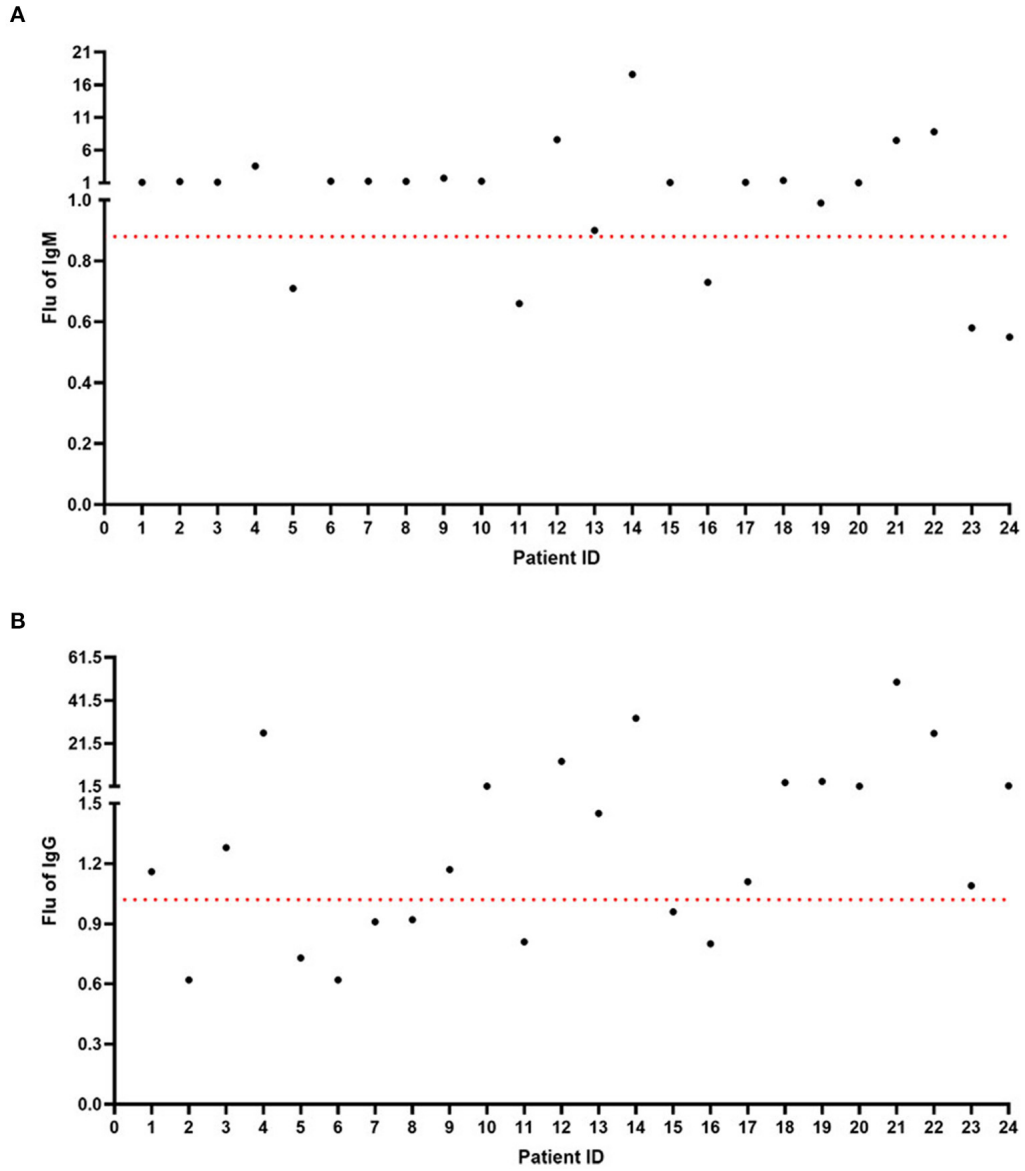
IgM and IgG detection among the 24 patients with positive COVID-19 nucleic acid results. **(A)** The IgM-value of 19 patients was more than 0.88 U/L. **(B)** The IgG-value of 16 patients was more than 1.02 U/L.

According to the nucleic acid test results, we performed IgM and IgG detection through the Diagreat company. As shown in Figure 1A, among the 33 patients with negative COVID-19 nucleic acid results, the IgM value of 20 patients was more than 0.88 U/L, and the positivity rate was 60.61%. As shown in Figure 1B, the IgG-value of 15 patients was more than 1.02 U/L, and the positivity rate was 45.45%. As shown in Figure 2A, among the 24 patients with positive COVID-19 nucleic acid results, the IgM value of 19 patients was more than 0.88 U/L, and the positivity rate was 79.17%. As shown in Figure 2B, the IgG-value of 16 patients was more than 1.02 U/L, and the positivity rate was 66.67%.

### Comparison of Exposure Times for NAAT and Serological Tests

The time from the first exposure to infection to nucleic acid testing ranged from 1 to 34 days. One patient had a negative nucleic acid result 34 days after exposure; however, the IgM detection result was positive. The results were partly different from the current understanding that the median incubation period for COVID-19 is 3 days, with a minimum of 0 days and a maximum of 24 days.

**Figure 3:**
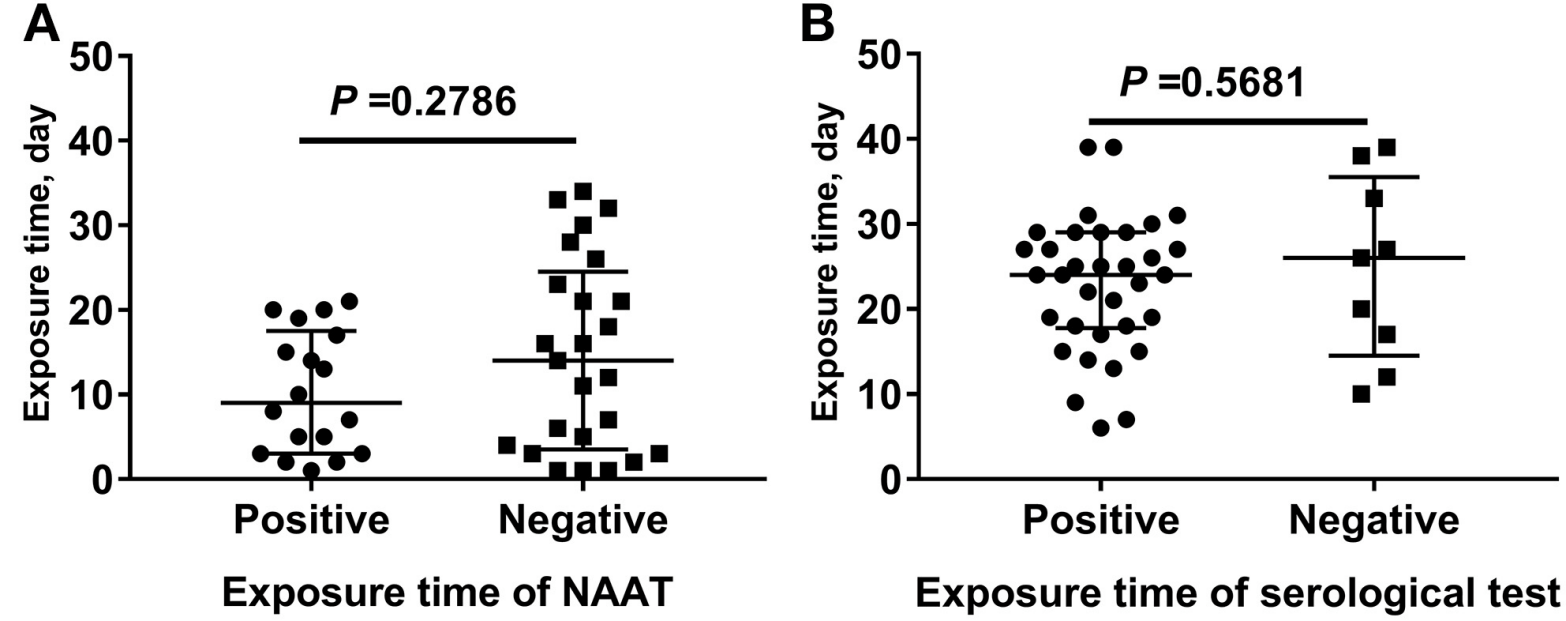
Comparison of exposure times for NAAT and serological tests. **(A)** The SARS-CoV-2 exposure time between the nucleic acid amplification test (NAAT) positive group and the NAAT negative group. **(B)** The SARS-CoV-2 exposure time between the serological test positive group and the serological test negative group.

A comparison of exposure times for NAAT and serological tests (Figure 3) revealed no statistically significant differences in SARS-CoV-2 exposure times between the NAAT positive group and the NAAT negative group. The results were the same as those of the serological tests.

### Combination of IgM and IgG Detection of COVID-19

**Figure 4:**
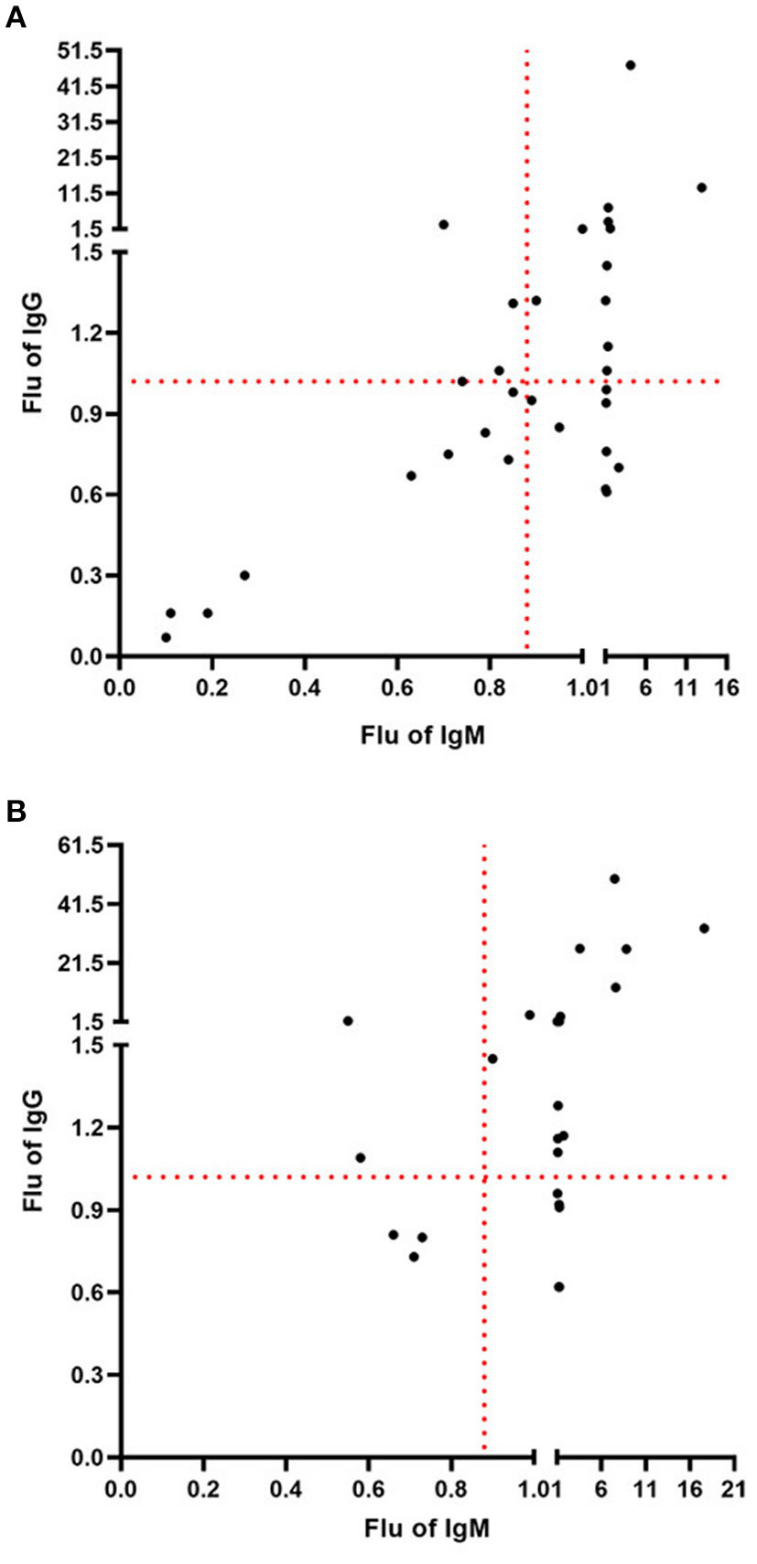
Combination of IgM and IgG detection of COVID-19. **(A)** The positive diagnostic rate with a combination of IgM and IgG detection for 33 patients with negative COVID-19 nucleic acid test results was 72.73%. **(B)** The positive diagnostic rate with a combination of IgM and IgG detection for 24 patients with positive COVID-19 nucleic acid test results was 87.50%.

Figure 4A shows the combination of IgM and IgG detection of COVID-19. Among the 33 patients who had a negative nucleic acid test, the percentages of IgM(+)IgG(+), IgM(–)IgG(+), IgM(+)IgG(–), and IgM(–)IgG(–) were 36.37, 12.12, 24.24, and 27.27%, respectively. The positive diagnostic rate with a combination of IgM and IgG detection for 33 patients with negative COVID-19 nucleic acid test results was 72.73%. Compared with a negative nucleic acid test and IgM and IgG single detection, the combination of IgM and IgG detection had a significantly higher positivity rate (*P* < 0.01). As shown in Figure 4B, in the 24 patients with a positive nucleic acid test, the combination of IgM and IgG detection of COVID-19 resulted in percentages of IgM(+)IgG(+), IgM(–)IgG(+), IgM(+)IgG(–), and IgM(–)IgG(–) of 62.50, 8.33, 16.67, and 12.50%, respectively. The positive diagnostic rate of a combination of IgM and IgG detection for 24 patients with COVID-19 negative nucleic acid test results was 87.50%. Compared with a nucleic acid positive test and IgM and IgG single detection, the combination of IgM and IgG also had a significantly higher positivity rate (*P* < 0.01).

### CT Scan of Two Special Patients

**Figure 5:**
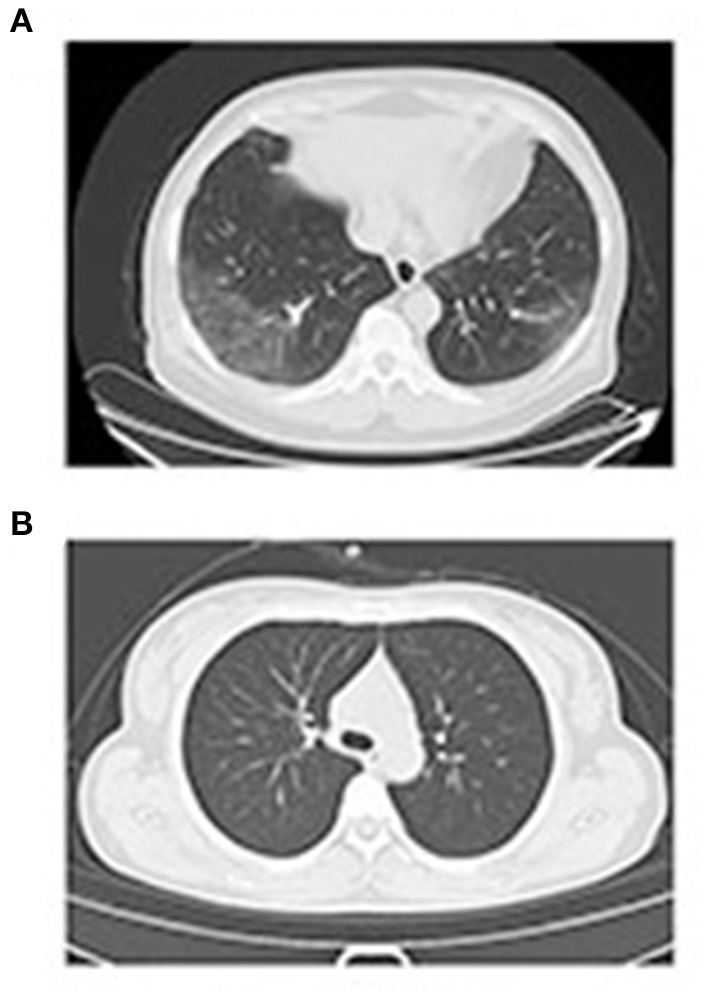
Patient case CT scan. **(A)** For patient 55, the nucleic acid detection result was negative, but the IgM and IgG results were positive. In the lower regions of both lungs, large fuzzy shadows, GGO, and a slightly fan-shaped distribution were observed. **(B)** In patient 19, the nucleic acid detection result was positive, but the IgM and IgG results were negative, and no clear lesions were found in the lungs.

A patient (number 55, female, 62 years old) presented with fatigue and fever on February 19, 2020. The nucleic acid detection results of pharyngeal swabs were negative on February 19 and 20. Serum IgM and IgG results were positive, and the values were 7.49 and 50.03 U/L. Chest computed tomography (CT scan) was performed on February 20, 2020, and the results are shown in Figure 5A. Characteristic changes in positive imaging findings were observed. In the lower region of both lungs in this patient, the CT scan showed large fuzzy shadows and ground-glass opacity (GGO), and a slightly fan-shaped distribution. Clinical symptoms, serological tests, and characteristic changes in the CT imaging of this patient were consistent.

Another patient (number 39, female, 35 years old) presented with cough and diarrhea on February 3, 2020. The nucleic acid detection results of pharyngeal swabs were positive on January 28, 2020. Serum IgM and IgG results were negative, and the values were 0.71 and 0.73 U/L. A CT scan was performed on February 8, 2020, and the results are shown in Figure 5B. The CT results showed no clear lesions in both lungs. Serological tests and characteristic changes in CT imaging of this patient were consistent.

### Association of CT Results With PCR Results and With Serological Results

**Figure 6:**
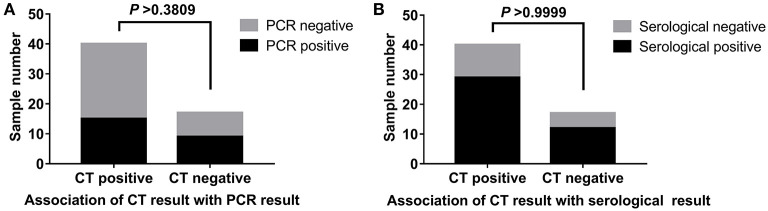
Association of CT results with PCR results and with serological results. **(A)** The proportion of positive NAAT between the positive and negative CT imaging groups. **(B)** The serological results between the positive and negative CT imaging groups.

Chest computed tomography (CT) scans of patients were assessed in the hospital. Characteristic changes of positive imaging findings included the following: multiple small patches and ground-glass opacity in both lungs, and infiltration and consolidation of opacity. Associations of CT results with PCR results and with serological results are shown in Figure 6. We found no statistically significant difference in the proportion of positive NAAT between the positive and negative CT imaging groups. The results were the same as those for the serological tests.

## Discussion

In our study, the COVID-19 nucleic acid positivity rate in the 57 cases of suspected COVID-19 was 42.10%. The nucleic acid test results may be false negative or false positive. Throat swab samples were collected in our study, and prior studies have demonstrated that sample location is a very important factor in nucleic acid detection. In one study, researchers analyzed a total of 72 nasal swabs and 72 throat swabs and obtained 9 consecutive samples from each patient; they detected a higher viral load shortly after the onset of symptoms and found that the viral load in the nose was higher than that in the throat (11). Studies have confirmed that bronchoalveolar lavage fluid specimens show the highest positive rates (14 of 15; 93%), followed by sputum (72 of 104; 72%), nasal swabs (5 of 8; 63%), fibrobronchoscope brush biopsies (6 of 13; 46%), pharyngeal swabs (126 of 398; 32%), feces (44 of 153; 29%), and blood (3 of 307; 1%) (12, 13). A systematic review and meta-analysis of SARS-CoV-2 using RT-PCR in different types of clinical specimens also revealed that the lower respiratory tract had the highest positive rate followed by rectal swab then sputum (14). In order to increase the accuracy of detection, one nasopharyngeal swab and one oropharyngeal swab are suggested to be collected into a single collection tube at the same time (15). Moreover, in clinical practice, when collecting throat swab specimens, medical staff must wear protective clothing. In this study, different medical staff performed the sample collection, and this procedure might have affected the nucleic acid detection results. Specimen collection operators should be trained (15) and an amplification reagent with “internal standard” was suggested to check whether the sample is qualified (16). As shown in Figure 5A, the nucleic acid detection results for patient 55 were negative, but the IgM and IgG results were positive. Characteristic changes in positive CT imaging findings were found. Finally, because of potential laboratory contamination, the positive results might have been false positive. As shown in Figure 5B, the nucleic acid detection result for patient 19 was positive, but the IgM and IgG results were negative, and the CT results showed no clear lesions in both lungs. One study has analyzed 126 German citizens who left Wuhan and were required to pass screening for clinical signs of infection: two passengers' nucleic acid tests were positive after quarantine for 14 days, but the two patients did not develop symptoms. The researchers reconfirmed the results through other methods. The results suggested that people with no fever, no symptoms, or only mild symptoms of infection may ignore their potential infectivity (17). Laboratory results for a COVID-19 nucleic acid positive group of 31 patients and a negative group of 23 patients have been found to be mainly characterized by diminished lymphocyte counts and elevated C-reactive protein levels; except for dyspnea, significant differences were observed in the clinical characteristics of the COVID-19 nucleic acid negative and positive groups (18). Studies have demonstrated that the clinical features of COVID-19 nucleic acid positive and negative patients are similar (19). We also studied the associations among CT results, PCR results, and serological results (Figure 6), and found no statistically significant difference in the proportion of positive NAAT between the positive and negative CT imaging groups. The results were the same as those for the serological tests. CT scans for the diagnosis of COVID-19 lack specificity (20). Faster and more accurate methods are urgently needed for the diagnosis of COVID-19. The positive diagnosis rates with a combination of IgM and IgG detection for patients with COVID-19 negative and positive nucleic acid tests were 72.73 and 87.50%, respectively. For 1 year, the SARS-CoV-2 antibody method including an enzyme-linked immunosorbent assay, a rapid immunochromatographic assay, and a chemiluminescent immunoassay has been applied and its clinical value is being evaluated (21). A study revealed that IgM/IgG-based detections can also result in false positive/false negative outcomes (22).

Some limitations are present in our study. First, the relatively small sample size, differences in IgM and IgG antigen binding sites, and differences in COVID-19 nucleic acid test design may have resulted in bias of the results. Second, the positivity rate of IgM and IgG tests may have been affected by the different times between viral exposure and detection. Earlier and different times should be examined, and the detection values of IgM and IgG should be further validated. Third, the detection value of IgM and IgG should be followed up in a future study.

## Conclusion

Compared with nucleic acid detection, IgM and IgG detection may provide a quick, simple, and accurate detection method for suspected COVID-19 cases. IgM and IgG antibody detection can identify suspected cases with negative nucleic acid tests. Diagnostic accuracy of COVID-19 might be improved by nucleic acid testing in patients with a history of epidemic disease or with clinical symptoms, as well as CT scans when necessary, and serum-specific IgM and IgG antibody testing after the window period.

## Data Availability Statement

The raw data supporting the conclusions of this article will be made available by the authors, without undue reservation.

## Ethics Statement

The studies involving human participants were reviewed and approved by the Ethics Committee of Shenzhen Hospital, Southern Medical University (NYSZYYEC20200009). Written informed consent for participation was not provided by the participants' legal guardians/next of kin because: This is a retrospective study and the data were anonymous, so the requirement for informed consent was therefore waived.

## Author Contributions

XJ, YT, JunW, KH, and YL contributed to the study design. JunlW and HZ contributed to data collection. JL and HH contributed to the collection of clinical specimens. XJ, ZC, and LZ contributed to experiments and data collection. PZ contributed to the data analysis. XJ, PZ, and YT contributed to the manuscript preparation. All authors contributed to the article and approved the submitted version.

## Conflict of Interest

The authors declare that the research was conducted in the absence of any commercial or financial relationships that could be constructed as a potential conflict of interest.
